# CARE: towards customized assistive robot-based education

**DOI:** 10.3389/frobt.2025.1474741

**Published:** 2025-02-21

**Authors:** Nafisa Maaz, Jinane Mounsef, Noel Maalouf

**Affiliations:** ^1^ Electrical Engineering Department, Rochester Institute of Technology, Dubai, United Arab Emirates; ^2^ Electrical and Computer Engineering Department, Lebanese American University, Byblos, Lebanon

**Keywords:** customized assistive robot-based education social robots, human-robot interaction, robot operating system (ROS), education, learning enhancement

## Abstract

This study proposes a novel approach to enhancing the learning experience of elementary school students by integrating Artificial Intelligence (AI) and robotics in education, focusing on personalized and adaptive learning. Unlike existing adaptive and intelligent tutoring systems, which primarily rely on digital platforms, our approach employs a personalized tutor robot to interact with students directly, combining cognitive and emotional assessment to deliver tailored educational experiences. This work extends the current research landscape by integrating real-time facial expression analysis, subjective feedback, and performance metrics to classify students into three categories: Proficient Students (Prof.S), Meeting-Expectations Students (MES), and Developing Students (DVS). These classifications are used to deliver customized learning content, motivational messages, and constructive feedback. The primary research question guiding this study is: Does personalization enhance the effectiveness of a robotic tutor in fostering improved learning outcomes? To address this, the study explores two key aspects: (1) how personalization contributes to a robotic tutor’s ability to adapt to individual student needs, thereby enhancing engagement and academic performance, and (2) how the effectiveness of a personalized robotic tutor compares to a human teacher, which serves as a benchmark for evaluating the system’s impact. Our study contrasts the personalized robot with a human teacher to highlight the potential of personalization in robotic tutoring within a real-world educational context. While a comparison with a generic, unpersonalized robot could further isolate the impact of personalization, our choice of comparison with a human teacher underscores the broader objective of positioning personalized robotic tutors as viable and impactful educational tools. The robot’s AI-powered system, employing the XGBoost algorithm, predicts the student’s proficiency level with high accuracy (100%), leveraging factors such as test scores, task completion time, and emotional engagement. Challenges and learning materials are dynamically adjusted to suit each student’s needs, with DVS receiving supportive exercises and Prof. S receiving advanced tasks. Our methodology goes beyond existing literature by embedding a fully autonomous robotic system within a classroom setting to assess and enhance learning outcomes. Evaluation through post-diagnostic exams demonstrated that the experimental group of students using the AI-robot system showed a significant improvement rate (approximately 8%) over the control group. These findings highlight the unique contribution of this study to the field of Human-Robot Interaction (HRI) and educational robotics, showcasing how integrating AI and robotics in a real-world learning environment can engage students and improve educational outcomes. By situating our work within the broader context of intelligent tutoring systems and addressing existing gaps, this study provides a unique contribution to the field. It aligns with and builds upon recent advancements, while offering a distinct perspective by incorporating robotics to foster both academic and emotional engagement.

## 1 Introduction

Over the past decade, researchers have studied people’s attitudes toward Socially Assistive Robots (SAR) in various contexts, including education (Park et al., 2010; Flandorfer, 2012; Johnson et al., 2014; Hinks, 2021; Dou et al., 2021). Studies have shown that people generally prefer teaching assistant robots over independent robot teachers in educational settings, and they also prefer one-to-one learning conditions with a robot rather than a group or classroom setting (Reich-Stiebert and Eyssel, 2015; Baxter et al., 2017). The efficiency of the SAR as an educational tool was evident since the late 2010s (Silvera-Tawil and Roberts-Yates., 2018; Umbrico et al., 2020; Rudovic et al., 2018; Belpaeme et al., 2018; Baxter et al., 2017; Jones and Castellano, 2018), with many studies focusing on using these robots to assist students with special educational needs such as autism and hearing disabilities (Silvera-Tawil and Roberts-Yates, 2018; Rudovic et al., 2018; Taheri et al., 2021; Ismail et al., 2021; Uluer et al., 2021; Wullenkord and Eyssel, 2020; Singh et al., 2022).

The embodiment of the robot serves as a pivotal factor, particularly in the early stages of education, fostering enhanced student participation in the learning experience (Conti et al., 2020; Ali et al., 2022). Given the prevalent use of technology in children’s daily lives, acclimating to the presence of a robot in the educational setting is anticipated to be seamless and well-received. The social robot is designed not only to interact with students but also to autonomously retrieve psychological indicators through analysing facial expressions of students and their subjective feedback. Leveraging this autonomous capability, coupled with the systematic recording of students’ scores, our aim is to empower the robot to effectively tailor educational materials based on individual learning profiles.

Integrating AI capabilities into robots to assist students and teachers has the potential to revolutionize the academic industry. However, this requires a thorough understanding of students’ diverse educational needs and cognitive performance. It is crucial to carefully select or create educational resources that provide relevant content and are presented in an engaging manner to support students’ learning progression, leaving no gaps in their knowledge and building upon their existing understanding. Teachers have a difficult but crucial responsibility to identify the unique differences among students in a classroom, and then work to address their weaknesses and build upon their strengths (Soysal et al., 2022; Manzone and Nyberg, 2022).

Recent advancements in intelligent tutoring systems (ITS) have further explored this potential. For example, Zhang et al. (2024) conducted a systematic review of intelligent tutoring robots, emphasizing their ability to enhance student learning through adaptive and personalized approaches. Building on this foundation, our study integrates real-time emotional and cognitive assessments into robotics, providing a unique contribution to the ITS field by addressing the challenges of personalized engagement and tailored educational support in real-world classroom environments.

**FIGURE 1:**
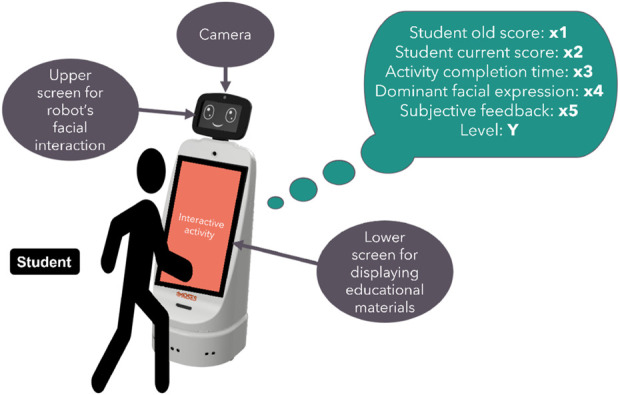
The robot Duet is used to assess and support students.

This paper proposes a novel method for assessing a student’s level in mathematics using multiple indicators and the extreme gradient boosting (XGBoost) model for classification (Chen et al., 2015). The indicators used are both subjective and objective, whereas teaching is conducted through interactive activities with the assistance of Duet, the interactive robot from Marses Robotic Solutions, as shown in Figure 1. For the specific task at hand, the use of any robotic system necessitates an interactive screen and a camera for enhanced user interaction and control.

To assist with the level prediction, the robot captures the student’s facial expressions using its upper camera. In addition, the robot is equipped with two screens - one that displays the robot’s facial expressions, and another touch screen that is used to display messages and facilitate interactive tasks.

The rest of the paper is organized as follows: Section 2 provides a review of related work, while Section 3 details the materials and methods used in the experiment, including the experimental setup and data description. In Section 4, the results are presented and analyzed. Finally, Section 5 concludes the study and offers suggestions for future work.

## 2 Related work

The prevalence of social robots increased during the early 21st century due to advancements in AI and robotics technology. Nevertheless, the idea of social robots can be traced back to the initial stages of research on robotics and AI in the early 1950s (Nørskov, 2017). Since then, the development of social robots has been an important milestone in the field of robotics, with applications in various domains such as healthcare, entertainment, and education. In education, social robots have been recognized as a valuable tool for enhancing the learning experience of students in a multitude of ways. These include increasing student engagement and motivation, improving learning experiences, and enabling the development of new teaching techniques and approaches.

The use of robots in educational settings has demonstrated potential to enhance both learning outcomes and social behaviors. To optimize robotic tutoring, incorporating proactive behavior is critical. Kraus et al. (2023) explores how robotic tutors can leverage students’ cognitive-affective states to initiate proactive tutoring dialogues, particularly in concept-learning scenarios. The study observed that robotic tutors intervening during negative states such as frustration and confusion helped maintain student focus. However, it also found that excessive proactivity could harm trust, emphasizing the need for balanced and context-sensitive proactive assistance to improve the effectiveness of robotic tutoring systems. Similarly, Hasan et al. (2020) investigates the shift from ITS to Affective Tutoring Systems (ATS). While ITS rely on cognitive data to personalize learning, ATS integrate emotional intelligence to detect and respond to students’ emotions using tools like facial recognition and physiological sensors. The paper identifies challenges such as ethical concerns, technological limitations, and cultural variability in emotion expression, alongside open issues regarding ATS integration in diverse educational settings. This research is particularly relevant in assessing how ATS-equipped robots can emulate or complement the emotional engagement provided by human tutors, further contributing to the development of affective robotic tutoring systems.

Baxter et al. (2017) opted to use the NAO robot as a peer learner in order to create a more encouraging learning environment for children, as it was believed that students are more comfortable dealing with a companion who makes mistakes. The study focused on observing two groups of learners: one group interacted with a personalized robot peer, while the other group interacted with a non-personalized version. The personalized robotic group showed more positive learning indicators. However, the main drawback of the NAO robot is its inability to engage with students using facial expressions, as its face remains static. In our work, the robot serves as a social interactive platform, placing emphasis on engaging the student in the learning process. Regular feedback is gathered systematically to ensure the establishment and maintenance of a social connection between the robot and the learner.

Pareto et al. (2022) investigated the use of a robot as a “tutee” or student in the “learning-by-teaching” method. The success of this approach was reliant on the quality of the interaction between the tutor and tutee. The authors assessed the level of engagement exhibited by the child when the tutee was a robot compared to when it was a younger child. The tutor was able to provide more accurate explanations and prevent misunderstandings with the child tutee due to being mindful of the robot’s speech recognition limitations. However, the robot posed more questions than a younger child, presenting a challenge to the tutor and leading to enhanced tutor’s critical thinking skills. The questions asked by the robot in our work rely on the robot’s assessment of the learner. Considering the intellectual nature of the topic being evaluated, some questions may be repeated to strengthen the student’s understanding.

Eva is an example of a human-like tutor robotic head that reads emotions through facial expressions and speech. It is capable of mimicking human facial expressions through animation software and the Facial Action Coding System (FACS) (Lazzeri et al., 2018). Employing a fully robotic body for Eva holds promise for significant advancements in the field of education, which would be beneficial for our particular application.

Another example is the Softbank Pepper humanoid robot, which was used to support the wellbeing of students diagnosed with autism in a school (Lemaignan et al., 2022). Pepper was left to operate autonomously in the school corridor, where it met different students and interacted with them. The study showed a variety of responses among the study group, including teachers who recommended that the interaction with the robot should be more personalized. Consequently, our study aims to gather personalized indicators, potentially expanding to accommodate learners with special educational needs.

Rudovic et al. (2018) have estimated the engagement levels of a number of children with autism (valence, arousal, and engagement) during their therapy sessions, which were led by a therapist with the assistance of NAO. The robot was controlled by the therapist, and the child was required to wear a wristband equipped with physiological sensors to enhance engagement levels. The authors prioritised studying engagement levels over establishing a social bond between the robot and the child during therapy. In our work, we contend that fostering a social connection between the robot and the learner is pivotal in shaping the learning process.

Esfandbod et al. have studied the feedback of students with hearing impairment on APO, a reading lips social robot (Esfandbod et al., 2022). The robot’s objective is to enhance the lip-reading skills of the target group. According to the results of the experiment, the robot received higher acceptance than a recorded lip-syncing video. As a result of using an LCD screen to display lip-synchronized words, the illustration was limited to a two-dimensional representation. The model we are proposing offers customized activities tailored to learners’ specific needs.

In another study conducted by de Souza Jeronimo et al. (2022), NAO and Zenbo social robots were employed to teach children music, wherein the robots were able to recognize the sound of a guitar string and teach the children how to properly tune the guitar. The study aimed to assess their favorite robot embodiment. Most participants preferred using Zenbo over NAO. However, the comparison was not completely equitable as the quality of the videos used for teaching was not consistent between the two robots. In our suggested approach, the robot’s embodiment could vary as long as it is equipped with both a camera and an interactive screen to facilitate human-robot interaction.

Previous research has indicated positive outcomes in using social robots to enhance children’s learning abilities. Nevertheless, there are still certain limitations that need to be addressed. These include the robot’s limited ability to interact with students using facial expressions, the restricted range of measures the robot can use to assess user performance, and the underutilization of the data gathered by the robot. In light of these challenges, we developed a tutor robot that improves the ability to interact with students using facial expressions, expanding the range of measures to assess user performance, and enhancing the data utilization capabilities by employing powerful machine learning algorithms such as XGBoost to identify insights that can inform the teaching process and optimize the learning experience.

In the work of Karthigha and Akshaya (2022), the XGBoost algorithm achieved an accuracy of 95.01% in predicting human sepsis disease using multiple features such as the heart rate, pulse oximetry, temperature, systolic blood pressure, and a few others. The XGBoost outperformed other commonly used models including the decision tree, gradient boosting tree, and random forest. In another study conducted by Alhaddad et al. Alhaddad et al. (2022), the XGBoost algorithm achieved the highest accuracy (90%) in characterizing five possible undesired interactions between a child and a social robot. Moreover, the XGBoost algorithm demonstrated a better performance and required significantly less training than the random forest and support vector machines (SVM) algorithms due to its ability to handle sparse data and model complex relationships through gradient boosting techniques, which iteratively improve the accuracy of predictions. Unlike random forests that build trees independently and SVMs that require extensive parameter tuning, XGBoost optimizes both the model and computation speed by using a sparsity-aware algorithm and effective handling of missing values. For instance, in a student performance prediction task, XGBoost can quickly converge to an accurate model with fewer iterations, reducing computational costs and training time compared to the more resource-intensive random forest and SVM approaches. This could become particularly valuable if the model were to be adapted for more diverse student groups, including those with special needs. In such cases, incorporating additional features like physiological or vital sign data could enhance the model’s predictive validity and relevance. XGBoost’s efficiency with smaller datasets and complex features might make it particularly suited for such scenarios, offering both flexibility and high accuracy in more nuanced applications.

## 3 Materials and methods

In today’s education system, a noteworthy challenge arises in seamlessly blending cutting-edge tools with traditional methods to meet the evolving cognitive needs of students in our technology-driven era. The gap between conventional teaching methods and students’ desire for personalized, interactive learning experiences prompts a thorough examination. Our research focus revolves around the potential of incorporating robotics into education to address these challenges, aiming to bridge the gap between traditional teaching approaches and contemporary students’ preference for tailored and engaging learning encounters. Our work expands beyond the evaluation of academic performance to include the interpretation of psychological indicators and subjective feedback within a non-judgmental framework.

### 3.1 Level indicators description

The presented methodology is groundbreaking in its consideration of five level-indicators as metrics for assessing student performance. These indicators encompass both the psychological state and academic performance of the student, forming a comprehensive approach to performance evaluation. These indicators include: the dominant facial expression displayed by the student during their most recent interactive task with the robot, the score achieved by the student in that task, the student’s historical performance score, the duration of their interaction during the most recent interactive task with the robot, and the subjective feedback provided by the student regarding their satisfaction. Table 1 shows the variables listed under each indicator used to predict students’ levels.

**TABLE 1:** List of variables for the input indicators that are used to predict the performance level of a student (facial expression, old score, current score, completion time, and subjective feedback).

Facial exp	Current score	Old score	Completion time	Feedback	Level
happy	distinct	distinct	very early	positive	3
neutral	above average	above average	early	neutral	2
surprised	average	average	average	negative	1
sad	below average	below average	late		
angry	weak	weak	very late		
disgusted					
fearful					
contemptuous					

The Convolutional Neural Network (CNN) VGG13-PLD approach is used to analyze the student’s facial expression and identify one of eight possible emotions: neutral, happy, surprised, sad, angry, disgusted, fearful, or contemptuous (Barsoum et al., 2016). The CNN leverages the Bleedfacedetector package, which includes four different face detection models, to identify facial features and support facial expression analysis. By analyzing these detected features, the system calculates the likelihood of each potential emotion, allowing it to predict the dominant expression displayed by the learner.

As a second level-indicator, the student’s current score is captured from the most recent interactive activity completed with the robot using the Selenium web page automation tool. Based on the score, the student is classified into one of the following categories: distinct, above average, average, below average, or weak. The activities encompass a range of formats, including multiple-choice questions, fill-in-the-blank exercises, and matching tasks. This diverse array of question types is designed to assess and reinforce students’ understanding of the topic from different angles. By engaging with various formats, students can demonstrate their comprehension in multiple ways, allowing for a more thorough evaluation of their knowledge. Each activity is assigned a unique average score, determined by a professional consultant, who is an expert in mathematics teaching. The scoring is based on the activity format, the level of difficulty, and the number of questions in the activity.

To ensure accuracy, the assessment of a student’s proficiency level considers their previous score as a third level-indicator, in addition to their current score. By using the same scoring categories for both the previous and current scores, we aim to ensure a fair and impartial evaluation of the student’s comprehension. Total scores are not shown to students. Instead, incorrect answers are highlighted in red once the student submits their responses to the activity.

In addition to scores, the time required to complete an interactive activity is considered important for assessing the student’s mastery of the subject and their readiness for advancement. Based on the completion time, the student may be classified into one of the following categories: very early, early, average, late, or very late. An average completion time has been determined for each activity based on observations of the time students spent completing different types of tasks during the testing period of the experiment. The student’s completion time is classified into different categories based on the following criteria: very early completion is achieved if the student answers within 65% of the average time, early completion is achieved if the student answers within 70% of the average time, late completion is achieved if the student answers 30% above the average time, and very late completion is achieved if the student answers 50% above the average time. The classification of completion time into five groups and the calculation of the average time are based on observations made during students’ completion of various interactive activities.

Finally, the robot obtains subjective feedback by asking the student about their satisfaction and willingness to continue with the lessons. Pop-up messages are displayed at the end of each lesson to gather their feedback. The messages are presented in language that is easy for students to understand, and students respond by pressing one of the following buttons: positive, neutral, or negative. For example, the message “How do you find the lesson?” is followed by the options listed below:

• Button 1: Easy, I love the lesson with the robot!
•Button 2: Not easy, but I like it!
•Button 3: Hard, I do not enjoy the lesson!

**FIGURE 2:**
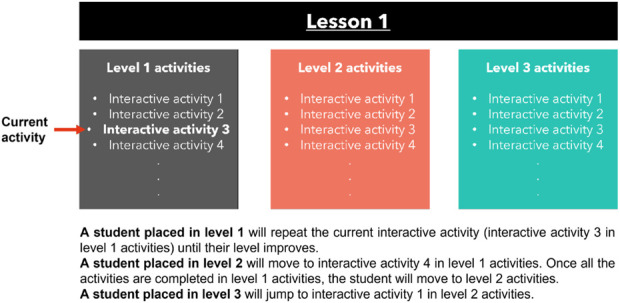
The proposed lessons structure and activities levels.

Based on the five previous level-indicators, the system classifies students into one of three proficiency levels: Proficient Students (Prof.S), Meeting-Expectations Students (MES), or Developing Students (DVS). Students classified as DVS (level 1) are required to repeat the same task until they improve and advance to the next activity. Students who are classified as MES (level 2) can proceed to the next activity within the lesson, following the activities’ sequence, while students classified as Prof. S (level 3) have obtained the necessary knowledge and ability to move on to the next level within the same lesson, allowing them to skip the activities of the level they have already mastered. A more detailed depiction of the classification process is presented in Figure 2.

The robot possesses a library of lessons that can be modified and expanded. In the initial experimental testing phase, which lasted 3 days, the library consisted of three lessons. However, during the subsequent experimental stage that spanned over 2 weeks, the library was expanded to include seven lessons. All students start with a level 1 interactive activity at the beginning of every lesson, regardless of their academic proficiency. To become acquainted with the robot’s interaction, the student must complete three activities before being evaluated by the model if it is their first session. Otherwise, the student’s level is evaluated at the completion of the second activity to assess their fundamental knowledge required to proceed with the remaining part of the lesson. In subsequent activities of the same lesson, the student’s level is determined upon completion of each activity. The structure of Lesson 1 is depicted in Figure 2, which is consistent with the structure of all other lessons provided online by the Ministry of Education in UAE at Edushare platform. The proposed model uses data from the lessons to determine the student’s level.

### 3.2 Experimental procedure

The adopted experimental procedure employs a comprehensive approach to ensure personalized and effective learning for each student. This is achieved through the use of OpenCV’s face recognition tools to identify the student and access their previous lesson history. The student is welcomed and encouraged before starting their lesson activities, further enhancing the personalized nature of the learning experience.

During the activities, the student’s facial expression is analyzed using the VGG13 model to determine their current emotional state (Barsoum et al., 2016). The VGG13 model’s robustness to noise and light variations enhances its ability to function effectively under real-world conditions, ensuring reliable student identification and emotional state assessment. Emotional analysis is a critical component of our methodology, as it allows the system to provide tailored feedback and adjust lesson content in real time. This level of personalization is central to the study’s objectives and would not be achievable with simpler identification methods. The score and completion time are recorded to monitor the student’s progress. Furthermore, the student’s feedback is solicited to provide a more accurate evaluation of their understanding and proficiency level.

**FIGURE 3:**
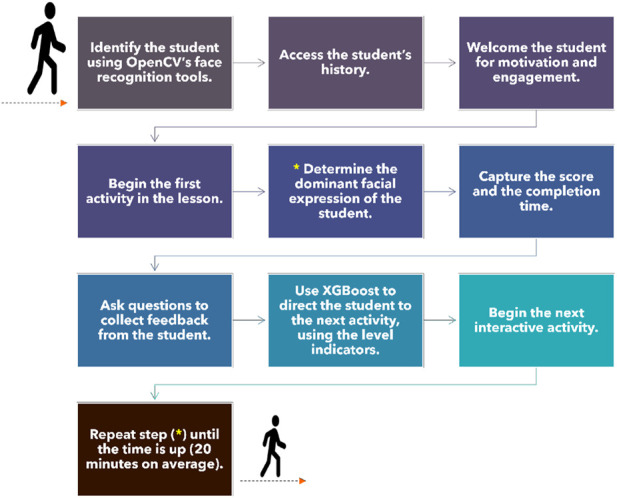
Different steps of Duet’s tutoring for a complete 20-minute session.

**FIGURE 4:**
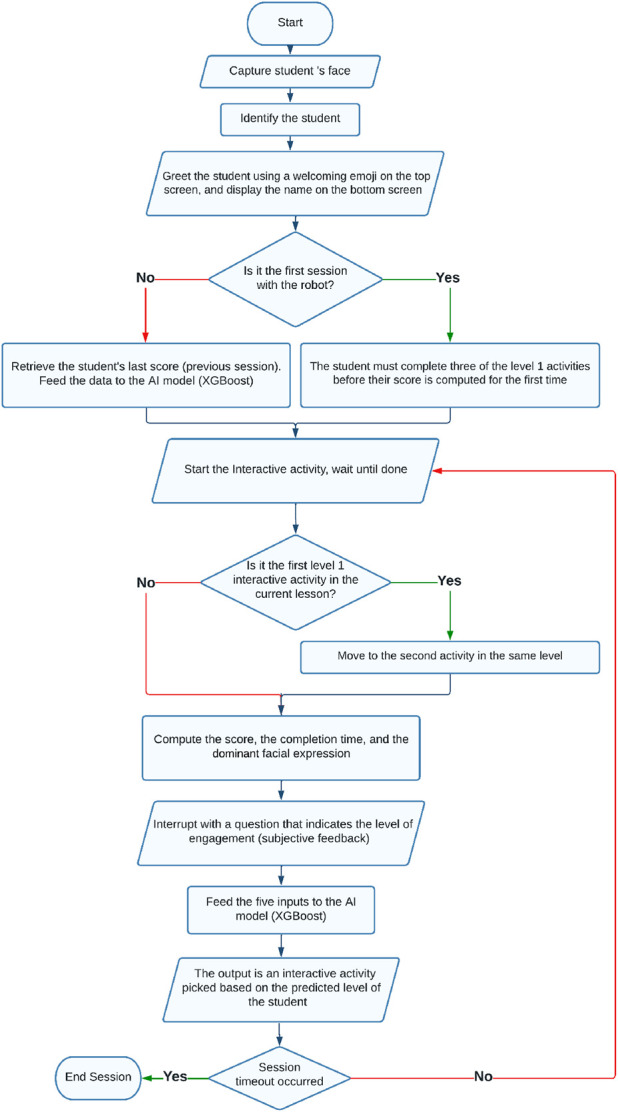
A flowchart illustrating the different steps of the proposed tutoring procedure.

Using the XGBoost gradient boosting model, a holistic assessment is performed based on the student’s facial expression, previous score, completion time, subjective feedback, and the score obtained in the last interactive activity. After the completion of each activity, the model updates the student’s level based on their performance, which is then used to assign appropriate material in the next activity. This process is continuously repeated until the end of the session, which typically lasts for 20 min. Figures 3, 4 illustrate the different stages of this procedure. The flowchart in Figure 4 outlines the different steps of the robot tutoring procedure, elucidating the specific functions attributed to XGBoost in assessing the student’s level and directing the students to the next activity tailored to match their needs.

#### 3.2.1 Data collection

The dataset used for the AI model was synthetically curated by generating various input (level indicators) and output (levels) combinations using Microsoft Excel. This process ensured the dataset encompassed all hypothetical data combinations of different inputs and output values. The initial dataset contained 9,000 entries. However, after a thorough review by two mathematics teachers, 6,064 entries were deemed unrealistic and removed, leaving a refined dataset of 2,936 entries. This refined dataset is non-repeating, consistent with its labels, and more representative of real-world student data.

The data filtering process was as follows: The first teacher reviewed the dataset to eliminate impossible combinations. For example, if a student’s scores were categorized as “weak” or “below average,” yet their placement level was listed as Level 3 (which is reserved for higher-performing students), such combinations were marked as unrealistic and discarded. Similarly, combinations where a student was categorized as “average” or “above average” but placed in Level 1—especially if their completion time was in the “early” or “on-time” range—were also considered invalid. After this initial filtering, the second teacher verified the decisions to ensure all remaining entries were valid and logically consistent.

To illustrate, Table 2 displays selected valid samples from the filtered dataset, showing consistent and realistic input-output mappings. Conversely, Table 3 provides examples of discarded entries, such as:

•A student with a “weak” score but assigned to Level 3.
•A student categorized as “above average” with an “on-time” completion but placed in Level 1.

The remaining 2,936 samples are unique and do not repeat across different labels. Each entry represents a logically consistent mapping between input indicators and the corresponding level, ensuring the dataset’s integrity for evaluating and predicting with the AI model.

**TABLE 2:** Selected samples of the filtered dataset.

Facial exp	Old score	Current score	Completion time	Feedback	Level
fearful	average	average	on time	neutral	2
happy	above average	distinct	very early	neutral	3
sad	weak	average	very late	positive	1
neutral	distinct	above average	very early	positive	3
neutral	average	average	on time	neutral	2
disgusted	weak	below average	on time	negative	1
angry	average	below average	late	neutral	1
sad	average	distinct	on time	neutral	2
angry	below average	average	early	negative	1
surprised	distinct	weak	late	negative	1
neutral	average	above average	on time	negative	2
neutral	average	distinct	very late	negative	2
contemptuous	below average	average	on time	negative	1

**TABLE 3:** Selected samples of the discarded data entries.

Facial exp	Old score	Current score	Completion time	Feedback	Level
fearful	average	average	very early	positive	3
surprised	below average	average	on time	positive	3
contemptuous	below average	average	on time	positive	3
neutral	below average	weak	late	positive	2
neutral	below average	weak	very early	neutral	2
happy	average	average	very early	negative	1
contemptuous	distinct	below average	early	positive	3
sad	weak	above average	late	neutral	3
fearful	weak	average	very early	neutral	3
neutral	weak	distinct	late	positive	2
neutral	weak	above average	late	positive	2
neutral	average	above average	early	negative	1
neutral	average	above average	on time	negative	1

### 3.3 Machine learning model

While the VGG13 deep neural network is used for facial expression recognition, the XGBoost model is employed to evaluate the student’s level. The VGG13 model is trained on a set of labeled images depicting expressions from different age groups, genders, and races. The model architecture comprises ten convolution layers, each interposed with max pooling and dropout layers. The first layer processes the input, followed by two convolution layers utilizing 64 3 × 3 kernels. After the max pooling layer, a dropout layer with a 25% dropout rate is introduced. This pattern is repeated with different numbers of convolution layers and kernels. The model also includes two dense layers with 1,024 hidden nodes each, followed by a 50% dropout layer after each dense layer. Finally, a softmax layer is added after the final dense layer to generate the output.

Data is prepared for machine learning by converting score and time classes into numerical values. Since facial expression categories lack an inherent order, one-hot encoding is used to represent the facial expression classes.

The XGBoost model is selected to classify the student’s level into one of three abilities. XGBoost, an acronym denoting eXtreme Gradient Boosting, employs an ensemble learning methodology featuring numerous constituent decision-making entities known as “decision trees.” Each decision tree within the ensemble iteratively updates the weightings assigned to its predecessors, thereby contributing to an optimized decision-making process. XGBoost encompasses three notable techniques: Boosting, Gradient Boosting, and Extreme Gradient Boosting:

1. Boosting: This machine learning approach combines several weak models—models that are marginally better than random chance—to forge a robust predictive model. Each subsequent model learns from the errors of the preceding ones, thereby incrementally boosting accuracy.
2. Gradient Boosting: A refinement of boosting, gradient boosting constructs models in a sequence, with each new model addressing the inaccuracies of its predecessors. It is akin to a collaborative team effort, where each new contribution builds on the collective learning from prior outcomes.
3. Extreme Gradient Boosting (XGBoost): Elevating gradient boosting, XGBoost introduces several improvements to enhance both efficiency and processing speed. It leverages parallel processing and tree pruning among other techniques, rendering it exceptionally versatile and powerful for various applications, including regression, classification, and ranking challenges.

One of the primary reasons for selecting XGBoost over a single decision tree is its ability to address the limitations of decision trees. A single decision tree tends to overfit the training data, especially in datasets with high variance or noise, as it tries to perfectly classify the training data by creating overly complex splits that do not generalize well to new, unseen data. Decision trees also struggle with representing complex, non-linear relationships or interactions between features due to their binary splitting nature. In contrast, XGBoost’s ensemble learning approach mitigates overfitting by combining multiple decision trees, each focusing on correcting the errors of its predecessor. This iterative process refines the model’s predictions, improving generalization and performance. Moreover, XGBoost incorporates advanced regularization techniques, such as L1 (lasso) and L2 (ridge) regularization, which prevent the model from becoming overly complex and help it generalize better. It also handles sparse or noisy data efficiently through sparsity-aware algorithms, making it robust in scenarios where features might be missing or incomplete. For example, in this dataset, dominant features like facial expressions and completion times exhibit complex interactions that a single decision tree cannot effectively model. XGBoost’s iterative learning mechanism captures these interactions and assigns importance to features adaptively, enhancing prediction accuracy. This capability also allows XGBoost to achieve high accuracy even with limited training data, unlike decision trees that may struggle with sparse data. Additionally, while SVM with an RBF kernel can model non-linear relationships, its decision boundaries are often complex and less interpretable than the decision trees in XGBoost. XGBoost combines the predictions of multiple trees to enhance robustness and accuracy, particularly in noisy datasets or those containing outliers. This interpretability makes XGBoost an excellent choice for educational applications where understanding model decisions is crucial.

The process begins with XGBoost taking various input features, such as facial expressions, previous scores, completion times, subjective feedback, and the latest activity scores. These features are preprocessed into a numerical format suitable for model training. During training, XGBoost builds an ensemble of decision trees iteratively. It starts with a base prediction and sequentially adds trees, each aiming to correct the errors of the previous ones by focusing on the residuals. For instance, if a student’s facial expression indicates confusion and their completion time is high, the model might predict a need for review material. Conversely, positive expressions and short completion times might suggest advancing the student. This iterative process continues until the model accurately predicts the student’s level, enabling personalized learning material assignment.

To validate the superiority of XGBoost in this context, additional experiments were conducted to compare its performance against decision trees. These experiments revealed that a random forest for example, failed to generalize well on test data due to overfitting and poor handling of complex relationships between features. XGBoost’s ability to iteratively refine predictions and handle sparse data was critical in achieving superior performance. Visualizations of feature importance further demonstrated how XGBoost effectively captures intricate patterns and interactions that decision trees miss. Such robustness and interpretability solidify XGBoost’s suitability for this application, as detailed in Section 4.

### 3.4 ROS structure

The proposed approach is implemented on the Robot Operating System (ROS) 2 - Humble distribution. ROS is an open-source middleware framework designed to facilitate the development of robotic software by providing services, tools, and a communication infrastructure that allow different components of a robot system to communicate with each other. The ROS model designed for the proposed work includes five nodes: the student identity node, which uses OpenCV’s face recognition to detect faces and VGG13 to verify the learner’s identity, the welcome node, the lesson planner node that plans the next activity by XGBoost, the subjective feedback node, and the main controller node - the lesson node. A single launch file is used to initiate all of the nodes. When the student’s identity node detects a face that matches one of the faces in the resources files (where all participant images are saved), it sends a string message to the lesson node with the student’s name. The lesson node then triggers the welcome node, which uses the student’s name to display a welcome message in the GUI. Once the welcome message is displayed, the lesson node progresses to the first activity in the lesson, where it calculates both the completion time and score. Then, the lesson node triggers the subjective feedback node, retrieves the student’s feedback, and transmits all the collected data to the lesson planner. The XGBoost model incorporated in the lesson planner uses this data to determine the student’s level and the next activity in the lesson. The activities are passed to the lesson node as links.

### 3.5 Participants and experimental setup

**FIGURE 5:**
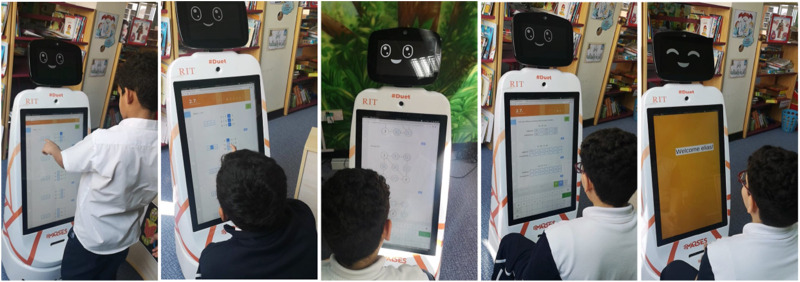
Students’ engagement and performance during the tutoring sessions with Duet.

Five fourth-grade students (9–10 years old) participated in the experiment, as shown in Figure 5. They were chosen by the mathematics teacher based on their scores in a pre-diagnostic exam, graded out of 15 marks, along with the teacher’s professional judgment. Similarly, a control group was formed using identical criteria of age, grade level, pre-diagnostic exam scores, and teacher’s professional judgment to facilitate comparison with the experimental group. The experimental group consisted of four boys and one girl, while the control group comprised of two girls and three boys. All participants were natives of the Middle East, originating from countries including the United Arab Emirates, Egypt, Jordan, and Yemen. They were citizens and expatriates who were born and raised in the United Arab Emirates. Additionally, all participants received their education within the framework of the country’s public school system. The experimental group received the instructional material through the robot, whereas the control group received the same instructional material but in a traditional classroom setting, with a mathematics teacher delivering the instruction. Table 4 illustrates the scores achieved by students in each group: 14 for the distinct student, 13 for the above-average student, 11 for the average student, 8 for the below-average student, and 5 for the student performing weakly.

**TABLE 4:** Diagnostic scores for the experimental and control groups.

Distinct Score	Above average score	Average score	Below average score	Weak score
14	13	11	8	5

For the experimental group, the experiment was conducted individually with one student at a time in the school’s library. The initial experimental stage lasted three days, during which the students were introduced to the robot and familiarized themselves with various question types, including drag and drop, fill-in-the-blanks, and reorder. Concurrently, an observer documented the system’s performance and identified any technical problems requiring attention or improvement. Three weeks following the completion of the initial stage, the experiment was repeated with the same students over a two-week period, which formed the second experimental stage. Meanwhile, the control group remained unaware of the experiment and continued attending regular math classes in a conventional classroom environment. They used tablets to complete lessons and activities, with their scores recorded for comparative analysis.

## 4 Results and discussion

The hyperparameters used to train the XGBoost model were carefully tuned to optimize performance. Key hyperparameters included a learning rate of 0.1, a maximum tree depth of 6, and a minimum child weight of 1. Additionally, the subsample ratio was set to 0.8, and the colsample˙bytree was 0.8, ensuring robust feature sampling. The number of boosting rounds was capped at 100 to balance training efficiency and performance. To evaluate the model’s ability to achieve 100% accuracy on the test set (98% of the data) while training on just 2% of the data, a stratified random sampling procedure was employed. This approach ensured that the training set was representative of the class distribution in the entire dataset. The train-test split was fixed to maintain consistency across experiments, and cross-validation was performed with five folds to assess performance variability. The cross-validation results indicated minimal performance variation, demonstrating the model’s stability and robustness in achieving high accuracy even with limited training data.

To further elucidate the model’s ability to learn the data structure with minimal training data, a comparative analysis was conducted using the XGBoost with a random forest and an SVM with RBF kernel.

**FIGURE 6:**
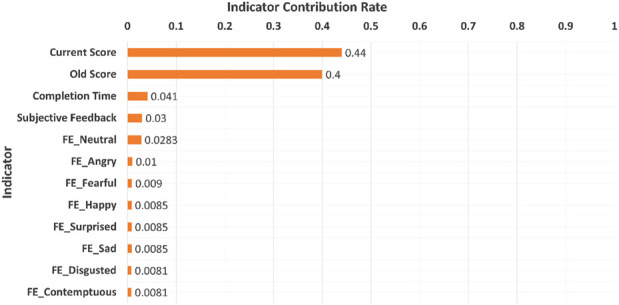
Indicators contribution rates to predict the student’s level.

Each of the three models achieved a perfect accuracy score of 100% when training the models on 70% of the dataset and testing them on the remaining 30%. These remarkable results can be attributed to the unique significance levels exhibited by each indicator used in the experiments. Specifically, the new score indicator emerged with the highest level of importance, with the old score and completion time following in order of importance, as shown in Figure 6. Determining each indicator’s importance is based on a comparison of impurity reduction or Gini index for each level indicator within the XGBoost training set.

As shown in Table 5, when training the three algorithms on reduced training sizes, all three achieved a 100% accuracy score at various performance levels. The SVM with RBF kernel achieved a 100% accuracy score when trained on 60% of the dataset. However, the random forest achieved a 100% accuracy score with only 19% of the dataset, while XGBoost outperformed them all by achieving a 100% accuracy score with only 2% of the dataset, making it the model of choice for our proposed method. When trained on such a limited dataset, the accuracy of SVM dropped to 79%, whereas random forest achieved an accuracy score of 84%.

**TABLE 5:** Minimum train and test sizes for 100% accuracy of the XGBoost, random forest, and SVM with RBF kernel models.

Test accuracy				
Train Size	Test Size	XGBoost	Random Forest	SVM
60%	40%	100%	100%	100%
19%	81%	100%	100%	86%
2%	98%	100%	84%	79%

The random forest, despite being an ensemble method of decision trees, failed to fully capture the intricate relationships between features for several reasons. Random forests can struggle with datasets where feature relationships are highly nonlinear or dependent on nuanced interactions. While the method reduces the risk of overfitting compared to a single decision tree, it can still exhibit overfitting tendencies in scenarios with imbalanced data or insufficient diversity in the training set. This issue can lead to overly complex trees within the ensemble, which collectively fail to generalize well to unseen data. For example, while the random forest relied heavily on dominant features such as facial expressions and completion times, it failed to integrate these features effectively with secondary attributes like subjective feedback. On the other hand, although the SVM with RBF kernel can model nonlinear relationships, performance is highly sensitive to hyperparameter tuning. Moreover, an RBF-based decision boundary is relatively opaque, making it more challenging to understand exactly how the model arrives at its predictions. In contrast, XGBoost’s ensemble learning and iterative corrections allowed it to model these complex relationships more effectively, highlighting its superiority in this context.

Therefore, our contribution is not simply using XGBoost as a powerful algorithm “off-the-shelf,” but rather demonstrating how and why it uncovers the underlying relationships in the data even with minimal training samples, where simpler or more interpretable models (like a random forest or an SVM) either underfit, require extensive tuning, or fail to capture the key feature interactions.

The data collected from the students was exported to a CSV file. Table 6 presents the results of the initial experimental stage, where the students’ names were replaced with a digit that denotes their rank within the group, ranging from 1 to 5.

**TABLE 6:** Data collection during the testing period of the first experimental stage.

name	Timestamp	Lesson	Last score	Time spent	Feedback	Expression
Stu-3	1670225305	1	weak	late	positive	Neutral
Stu-4	1670227196	1	weak	very late	positive	Neutral
Stu-1	1670229970	1	above average	very early	positive	Neutral
Stu-2	1670230327	1	weak	on time	positive	Neutral
Stu-1	1670310026	2	distinct	very early	positive	Neutral
Stu-3	1670312330	1	average	early	positive	Neutral
Stu-4	1670313014	1	distinct	on time	positive	unknown
Stu-2	1670314423	1	distinct	early	positive	Neutral
Stu-3	1670396295	2	distinct	very early	positive	unknown
Stu-5	1670398207	1	weak	very early	positive	Happy
Stu-2	1670402449	2	distinct	very late	positive	unknown
Stu-4	1670403127	2	distinct	very early	positive	Neutral

The testing sessions were conducted after the students had studied the topics relevant to the experiment and were preparing for the final mathematics exam of the term. These sessions provided an opportunity for the students to practice for the exam. Throughout the tutoring sessions, it became apparent that the students’ performance improved with increased interaction with the tutor robot. The data presented in Table 6 indicates that Stu-1 progressed from above average in the initial tutoring session to distinct in the following session. Stu-2 and Stu-4 also improved from a weak performance level in the first session to a distinct one in the subsequent sessions. Stu-3, initially classified as weak, advanced to an average level in the second session and eventually reached a distinct performance level in the last session. Although Stu-5 completed the interactive activities quickly in their only session, their low scores resulted in an evaluation of weak performance.

The second experimental stage began 3 weeks after the first experiment and lasted for 2 weeks, with the same students but with new lessons belonging to the following term’s curriculum. Prior to each session, the students were briefly introduced to the topics covered in the experiment. Students assigned to any of the three proficiency levels continued with the lesson activities, proceeding to the following lesson only if all levels in the current lesson had been accessed and assessed (Lesson Completed - LC). Each session, usually lasted between 20 and 25 min. Throughout the sessions, the students remained attentive and engaged, typically displaying neutral facial expressions. Occasionally, some students would look away or down, likely counting on their fingers while thinking, resulting in moments of unknown facial expressions. Additionally, since the VGG13 model was not explicitly trained to detect facial expressions in children, many emotions were not identified and were labeled as unknown.

It is commonly observed that students tend to perform better during the second evaluation of a lesson, which can be attributed to the practice received during tutoring sessions. The data shows that individual differences are considered, with Prof. S students demonstrating swift progress and understanding while DVS students required more time to practice and grasp the lesson content. Despite these differences, each student had a positive experience during all sessions.

Following each session, the mathematics teacher, who was also instrumental in refining the dataset, conducted an assessment of each student’s proficiency using five distinct level indicators. These assessments consistently aligned with those generated by XGBoost, indicating that students were appropriately placed at their respective levels during the robot tutoring sessions. The teacher’s evaluation thus confirmed the effectiveness of the XGBoost model. However, it is important to clarify that our proposed method operates autonomously without requiring direct input from the teacher. This means that the robot assesses the student without the teacher’s involvement while it is in operation. Nonetheless, we recommend taking the teacher’s insights into account when selecting activities based on the student’s proficiency level.

The effectiveness of the proposed method was validated by comparing the scores achieved by both the experimental and control groups and by calculating the percentage improvement between them.

To support our findings, we employed improvement rates calculated within each robotic session. The improvement rates were computed individually per lesson for each student, allowing us to evaluate differences in improvement rates between the experimental and control groups. Improvement rates were determined by our study’s professional consultant, the mathematics teacher, by analyzing scores from initial and concluding activities within each session. The improvement rate for each student was based on the difference between their starting score and their final score in a session, measured as a percentage of the starting score.

To address concerns about the potential interdependence of repeated measures within subjects, we acknowledge that improvement rates were calculated repeatedly for the same students across multiple sessions. This introduces a potential dependency structure that was not fully accounted for using the Mann-Whitney U test. While this test was initially chosen for its simplicity, we recognize its limitations and suggest that a mixed-effects model or a paired analysis could be explored in future analyses to address these dependencies explicitly.

Tables 7, 8 display the scores from both initial and concluding activities for each student per lesson. These tables illustrate the raw scores and calculated improvement rates for both experimental and control groups, supporting the comparative analysis of teaching methods. The data presented in these tables reveal that the experimental group demonstrated better improvement rates compared to the control group, highlighting the effectiveness of the robotic teaching sessions.

**TABLE 7:** Improvement rates per lesson for students in experimental group.

Student	Old score	New score	Improvement rate
Stu-1	89.31%	98.45%	10.23%
Stu-1	88.06%	97.03%	10.19%
Stu-1	88.33%	97.24%	10.09%
Stu-1	92.74%	100%	10.03%
Stu-1	89.29%	99.97%	11.96%
Stu-2	86.13%	93.12%	8.11%
Stu-2	87.13%	94.36%	8.3%
Stu-2	84.31%	91.47%	8.49%
Stu-3	78.13%	86.03%	10.11%
Stu-3	80.9%	89.31%	10.4%
Stu-3	79.62%	87.88%	10.38%
Stu-3	78.44%	85.27%	8.71%
Stu-4	75.75%	80.01%	5.62%
Stu-4	75.52%	79.43%	5.18%
Stu-5	69.91%	70.58%	0.96%
Stu-5	71.98%	72.15%	0.24%

**TABLE 8:** Improvement rates per lesson for students in control group.

Student	Old score	New score	Improvement rate
Stu-1′	92.25%	96.59%	4.71%
Stu-1′	89.69%	93.87%	4.66%
Stu-1′	89.95%	94.28%	4.81%
Stu-1′	90.4%	95.26%	5.38%
Stu-1′	89.73%	94.16%	4.94%
Stu-2′	86.2%	92.54%	7.36%
Stu-2′	84%	90.46%	7.69%
Stu-2′	83.28%	90.23%	8.35%
Stu-3′	78.05%	78.77%	0.92%
Stu-3′	80.5%	81.26%	0.95%
Stu-3′	81.49%	82.23%	0.91%
Stu-3′	81.19%	82.51%	1.62%
Stu-4′	73.86%	75.59%	2.34%
Stu-4′	76.28%	77.55%	1.66%
Stu-5′	72.43%	72.9%	0.65%
Stu-5′	70.65%	70.9%	0.35%

In summary, our analysis involved two distinct groups, each with a dataset of 16 improvement rate values, thereby facilitating a comprehensive comparative analysis. The average improvement rate is computed by averaging these improvement rates across all lessons, as illustrated in Table 9. In the experimental group, the average improvement percentage in scores peaked at 10.5%, while in the control group, it reached a maximum of 7.8%.

**TABLE 9:** The average score improvement of the experimental group compared to the control group for all lessons completed by the experimental group.

Student	% Improvement in exp. Scores	% Improvement in cont. Scores
Stu-1	10.5%	4.9%
Stu-2	8.3%	7.8%
Stu-3	9.9%	1.1%
Stu-4	5.4%	2.0%
Stu-5	0.6%	0.5%

Furthermore, students in both the experimental (Stu-x) and control (Stu-x’) groups took a post-diagnostic exam, the findings of which are compared to their diagnostic reference scores (Stu-x,x’), as displayed in Table 10. The exam focused on the same topics as those covered in the experiment. The results show that the experimental group students scored either equally or higher than the students in the control group, with a mean difference of approximately 1 point and an average improvement rate of about 8% higher than the control group, in comparison to their performance on the pre-diagnostic exam conducted before the experiment.

**TABLE 10:** Summative assessments exam scores of experimental (stu-x) vs. control (stu-x’) groups.

Student groups	x:1	x:2	x:3	x:4	x:5	Average improvement rate
Stu-x,x’	14	13	11	8	5	Reference scores
Stu-x	20	18	16	14	13	72.4%
Stu-x’	19	18	14	13	13	64.8%
			Mean difference		0.8 points	

Despite experiencing some intellectual challenges with certain activities, the students consistently maintained a favorable attitude toward the tutor robot across all lessons. The mathematics teacher observed that the students involved in the experiment displayed a greater interest in their mathematics classes and participated more actively in class activities after the experiment’s completion. According to the students’ feedback, while the robot piqued their interest, they expressed their preference for the robot to be more interactive by moving during sessions, incorporating games, or even providing tangible rewards for correct answers.

## 5 Conclusion and future work

In this work, we introduced a novel approach to personalized education through the development of a tutor robot to enhance the learning experience of elementary students. By integrating the XGBoost algorithm, our robot can assess students’ proficiency levels using a multifaceted set of indicators, such as scores and completion time for activities, facial expressions, and subjective feedback. This multifactorial evaluation allows the robot to tailor educational content specifically to each student’s needs and performance expectations, thereby offering a highly personalized and effective learning experience. The key contribution of our work lies in presenting a tutor robot that interacts with the students to personalize educational content by leveraging both their psychological and intellectual indicators. The proposed approach involves tailoring learning resources and experiences to match each student’s unique emotional reactions and cognitive evaluations, thereby addressing the varied needs, learning preferences and abilities of students, and promoting a more engaging and focused educational atmosphere. The integration of a physical robot into the customisation of educational content introduces a social dimension to student interactions, while also gathering and applying relevant data to enhance the learning process. The effectiveness of our method is supported by two factors: first, the experimental group displayed an improvement rate of 8% higher than the control group; second, the experimental group showed an increase in academic motivation and ability level.

Although our approach demonstrates significant potential, it is essential to acknowledge limitations that may affect its scalability and reliability in real-world settings. For instance, the use of facial recognition for identifying and evaluating students’ emotional states is known to be sensitive to environmental factors such as lighting conditions and camera angles. This sensitivity introduces potential error rates that could impact the accuracy of student-level predictions and the overall system performance. Future iterations of this research should include a detailed analysis of error rates in various real-world scenarios and investigate strategies to mitigate these limitations.

The experimental setup may have introduced confounds that affect the fairness of comparisons between the control and experimental groups. For instance, students in the robot group experienced a “tutorial session”, whereas students in the human teacher group did not. Additionally, the novelty effect of interacting with a robot may have influenced student engagement differently compared to the familiarity of a human teacher. These differences could skew the results, emphasizing the importance of addressing such factors in future experimental designs. Future work should include experiments that either equalize the conditions for both groups or isolate the impact of these variables to ensure a more rigorous evaluation of the system’s effectiveness.

Additionally, while the stated contribution of this work is the identification of relevant input features for personalization, the current algorithm groups students into three categories and delivers pre-scripted content rather than true user-specific personalization. This valid design choice could benefit from further exploration to clarify its implications and justify its practicality. Future research should aim to expand the personalization framework to incorporate finer-grained, dynamic adaptations tailored to individual student behaviors and needs. Moreover, direct comparisons between the interaction styles and pedagogical approaches of the robot and the human teacher, including factors such as prior familiarity with the teacher and the novelty of the robot, should be examined in depth to ensure a balanced evaluation.

Moreover, our study was conducted in a controlled environment with a relatively small sample size, which may not fully capture the complexities of diverse educational settings. Scaling the system to accommodate larger, heterogeneous groups of students will require robust testing and optimization. Future research should explore alternative methods for student identification, such as manual entry or wearable technologies, to enhance reliability while balancing practicality and user experience.

We also recognize that the real-time decision-making capabilities of the proposed algorithm, which are crucial for its practical applicability, remain untested in this study. Future work will focus on evaluating the system’s performance in live classroom settings under dynamic and variable conditions to ensure its effectiveness and reliability in real-world scenarios. Generating additional synthetic data and retesting the algorithm with real-world student data are essential steps to enhance the robustness of the validation process. These efforts will form a critical component of future work, employing data augmentation techniques, such as SMOTE (Synthetic Minority Over-sampling Technique), to supplement the dataset, while real-world trials will allow for a more comprehensive assessment of the algorithm’s performance and adaptability in diverse educational settings.

Moving forward, there is a potential to expand on the findings of this study by creating a platform that incorporates diverse and innovative learning materials to cater to learners of different proficiency levels. The platform should prioritize a user-friendly interface to enhance the overall learning experience. Additionally, we recommend developing a platform specifically tailored for the tutor robot, which seamlessly integrates with ROS to ensure accurate data collection. To complement the facial expression indicator, we suggest incorporating additional indicators collected through bio-signal sensors to enhance the student level prediction. Furthermore, the use of humanoid robots like Pepper, which are capable of engaging gestures, head, hands, and body movements, or a robotic head like Eva, can increase student engagement during tutoring sessions. Collaboration between education experts and robotics engineers is critical for the success of this research endeavor. Consequently, we highly recommend developing a comprehensive dataset encompassing all variables that could contribute to the evaluation of student performance.

## Data Availability

The raw data supporting the conclusions of this article will be made available by the authors, without undue reservation.
